# Supplementary material for: A quantum algorithm for evolving open quantum dynamics on quantum computing devices
Source: Sci Rep. 2020 Feb 24;10:3301. doi: 10.1038/s41598-020-60321-x (PMC7039952; doi:10.1038/s41598-020-60321-x)
Supplement: Supplementary file 1 — Supplementary information. [file 41598_2020_60321_MOESM1_ESM.pdf]

Supplementary information: A quantum algorithm for evolving open quantum dynamics on quantum computing devices

Zixuan Hu[1,2], Rongxin Xia[1] and Sabre Kais*[1]

1. *Department of Chemistry, Department of Physics, and Birck Nanotechnology Center, Purdue University, West Lafayette, IN 47907, United States*
2. *Qatar Environment and Energy Research Institute, College of Science and Engineering, HBKU, Doha, Qatar*
**Email:* kais@purdue.edu

This supplementary document supports the discussion in the main text by providing technical details. Section 1 provides the proof that each Kraus operator $\mathbf{M}_k$ is a contraction. Section 2 proves that $\tilde{\mathbf{O}}$ is a contraction and positive-semidefinite. Section 3 presents the modified algorithm for the initial state given in a general matrix form. Section 4 lists the quantum circuits used on the IBM Qiskit simulator and the IBM Q 5 Tenerife device.

1. Proof that each Kraus operator $\mathbf{M}_k$ is a contraction

As defined in the main text, an operator $\mathbf{A}$ is a contraction if it shrinks or preserves the norm of any vector such that the operator norm $\|\mathbf{A}\| = \sup \frac{\|\mathbf{A}\mathbf{v}\|}{\|\mathbf{v}\|} \le 1$. We have $\sum_k \mathbf{M}_k^\dagger \mathbf{M}_k = \mathbf{I}$, suppose an arbitrary one $\mathbf{M}_1$ is not a contraction, then for $\mathbf{M}_1$ there exists a vector $\mathbf{v}_1$ such that $\frac{\|\mathbf{M}_1\mathbf{v}_1\|}{\|\mathbf{v}_1\|} = \frac{\sqrt{\mathbf{v}_1^\dagger \mathbf{M}_1^\dagger \mathbf{M}_1 \mathbf{v}_1}}{\|\mathbf{v}_1\|} > 1$, then $\frac{\sqrt{\mathbf{v}_1^\dagger \sum_k \mathbf{M}_k^\dagger \mathbf{M}_k \mathbf{v}_1}}{\|\mathbf{v}_1\|} > \frac{\sqrt{\mathbf{v}_1^\dagger \mathbf{M}_1^\dagger \mathbf{M}_1 \mathbf{v}_1}}{\|\mathbf{v}_1\|} > 1$ because each $\mathbf{M}_k^\dagger \mathbf{M}_k$ is positive-semidefinite with $\mathbf{v}_1^\dagger \mathbf{M}_k^\dagger \mathbf{M}_k \mathbf{v}_1 \ge 0$. However because $\sum_k \mathbf{M}_k^\dagger \mathbf{M}_k = \mathbf{I}$ we also have $\frac{\sqrt{\mathbf{v}_1^\dagger \sum_k \mathbf{M}_k^\dagger \mathbf{M}_k \mathbf{v}_1}}{\|\mathbf{v}_1\|} = \frac{\sqrt{\mathbf{v}_1^\dagger \mathbf{v}_1}}{\|\mathbf{v}_1\|} = 1$. Hence by contradiction each $\mathbf{M}_k$ is a contraction.

2. Proof that $\tilde{\mathbf{O}} = \frac{\mathbf{O} + \mathbf{I}\|\mathbf{O}\|_{HS}}{2\|\mathbf{O}\|_{HS}}$ is a contraction and positive-semidefinite

To prove $\tilde{\mathbf{O}} = \frac{\mathbf{O} + \mathbf{I}\|\mathbf{O}\|_{HS}}{2\|\mathbf{O}\|_{HS}}$ is a contraction:

$$\left\|\tilde{\mathbf{O}}\right\| = \left\|\frac{\mathbf{O}+\mathbf{I}\|\mathbf{O}\|_{HS}}{2\|\mathbf{O}\|_{HS}}\right\| \le \frac{\|\mathbf{O}\|+\|\mathbf{I}\|\|\mathbf{O}\|_{HS}}{2\|\mathbf{O}\|_{HS}} \le \frac{\|\mathbf{O}\|_{HS}+\|\mathbf{O}\|_{HS}}{2\|\mathbf{O}\|_{HS}} = 1 \tag{1}$$

Where we have used the triangle inequality of the operator norm and the fact that $\|\mathbf{O}\| \le \|\mathbf{O}\|_{HS}$. Note we could have made $\tilde{\mathbf{O}} = \frac{\mathbf{O}+\mathbf{I}\|\mathbf{O}\|}{2\|\mathbf{O}\|}$ but the operator norm $\|\mathbf{O}\|$ is more difficult to calculate than the Hilbert-Schmidt norm $\|\mathbf{O}\|_{HS}$. To prove $\tilde{\mathbf{O}} = \frac{\mathbf{O}+\mathbf{I}\|\mathbf{O}\|_{HS}}{2\|\mathbf{O}\|_{HS}}$ is positive-semidefinite, let $\lambda_{\min}$ be the smallest eigenvalue of $\mathbf{O}$, then $|\lambda_{\min}| \le \|\mathbf{O}\| \le \|\mathbf{O}\|_{HS}$ and $\mathbf{v}^\dagger\mathbf{O}\mathbf{v} \ge \lambda_{\min}\|\mathbf{v}\|^2$ for any $\mathbf{v}$. Now we have for any $\mathbf{v}$:

$$\mathbf{v}^\dagger\tilde{\mathbf{O}}\mathbf{v} = \frac{\mathbf{v}^\dagger\mathbf{O}\mathbf{v}+\|\mathbf{O}\|_{HS}\|\mathbf{v}\|^2}{2\|\mathbf{O}\|_{HS}} \ge \frac{\left(\lambda_{\min}+\|\mathbf{O}\|_{HS}\right)\|\mathbf{v}\|^2}{2\|\mathbf{O}\|_{HS}} \ge 0 \tag{2}$$

Therefore $\tilde{\mathbf{O}}$ is indeed positive-semidefinite.

3. Modified algorithm for the initial state in a general matrix form

In this section we present the quantum algorithm that evolves $\rho(t)$ with the initial ρ given in a general matrix form:

$$\rho = \begin{pmatrix} \rho_{11} & \cdots & \rho_{1n} \\ \vdots & \ddots & \vdots \\ \rho_{n1} & \cdots & \rho_{nn} \end{pmatrix} \tag{3}$$

We first flatten ρ into a vector form:

$$\rho \to \mathbf{v}_\rho = \left(\rho_{11}, \ \dots, \rho_{1n}, \rho_{21}, \ \dots, \rho_{2n}, \ \dots\dots, \rho_{n1}, \ \dots, \rho_{nn}\right)^T \tag{4}$$

for which the norm of $\mathbf{v}_\rho$ is given by:

$$\left\|\mathbf{v}_\rho\right\| = \sqrt{\sum_{ij}\left|\rho_{ij}\right|^2} = \|\rho\|_{HS} = \sqrt{Tr\left(\rho^2\right)} \le 1 \tag{5}$$

where $\|\rho\|_{HS}$ is the Hilbert-Schmidt norm of the density matrix ρ. Eq. (5) connects the norm of $\mathbf{v}_\rho$ to the purity $Tr\left(\rho^2\right)$ which measures how much a mixture is ρ.

Now for each k in $\sum_k \mathbf{M}_k \rho \mathbf{M}_k^\dagger$ the $\mathbf{M}_k$ multiplying from the left is converted into $\mathcal{M}_k = \mathbf{M}_k \otimes \mathbf{I}$, and the $\mathbf{M}_k^\dagger$ multiplying from the right is converted into $\mathcal{N}_k = \mathbf{I} \otimes \bar{\mathbf{M}}_k$ where $\otimes$ stands for the Kronecker product, and the bar over $\mathbf{M}_k$ stands for complex conjugation. It is easy to verify that:

$$\mathbf{M}_k \rho \mathbf{M}_k^\dagger \xleftrightarrow{\text{equivalent}} \mathcal{N}_k \mathcal{M}_k \mathbf{v}_\rho \tag{6}$$

The core idea of this quantum algorithm is to represent $\mathbf{v}_\rho$ with a quantum state and then simulate the effects of $\mathcal{N}_k \mathcal{M}_k$ with quantum gates. Firstly $\mathbf{v}_\rho$ can be normalized and represented by an initial quantum state. In the main text we have proven each $\mathbf{M}_k$ is a contraction. It follows immediately that $\mathcal{M}_k = \mathbf{M}_k \otimes \mathbf{I}$ and $\mathcal{N}_k = \mathbf{I} \otimes \bar{\mathbf{M}}_k$ are also contractions by the norm property of the Kronecker product. To simulate $\mathcal{N}_k \mathcal{M}_k \mathbf{v}_\rho$ with unitary gates, we need two 2-dilations as in Eq. (8) in the main text by setting $\mathbf{A} = \mathcal{M}_k$ and $\mathbf{B} = \mathcal{N}_k$:

$$\mathcal{N}_k \mathcal{M}_k \mathbf{v}_\rho \xrightarrow{\text{unitary dilation}} \mathbf{U}_{\mathcal{N}_k} \mathbf{U}_{\mathcal{M}_k} \left(\mathbf{v}_\rho^T, 0, \ldots, 0 \right)^T \tag{7}$$

If $\mathbf{M}_k$ has the dimension n by n, then $\mathcal{M}_k$ and $\mathcal{N}_k$ are n^2 by n^2, and the 2-dilations $\mathbf{U}_{\mathcal{M}_k}$ and $\mathbf{U}_{\mathcal{N}_k}$ are $3n^2$ by $3n^2$. Now keeping with the main text we further decompose $\mathbf{U}_{\mathcal{M}_k}$ and $\mathbf{U}_{\mathcal{N}_k}$ into sequences of two-level unitary gates and count them towards the gate complexity. For $\mathbf{U}_{\mathcal{M}_k}$ the lower-triangular part contains $\frac{n^2 - n}{2} \cdot n$ non-zero elements from $\mathcal{M}_k$, n^3 non-zero elements from $\mathbf{D}_{\mathcal{M}_k}$, n^2 non-zero elements from $\mathbf{I}$, and the total count is $\frac{3n^3}{2} + \frac{n^2}{2}$. This count is the same for $\mathbf{U}_{\mathcal{N}_k}$, thus the total gate complexity to realize $\mathcal{N}_k \mathcal{M}_k \mathbf{v}_\rho$ is $3n^3 + n^2$ for each k. Note the classical complexity to realize $\mathbf{M}_k \rho \mathbf{M}_k^\dagger$ by two matrix multiplications is $4n^3 - 2n^2$ (using the naïve algorithm counting the number of multiplications and additions) for each k, which is of the same order of our proposed quantum algorithm, with a minor difference in the leading coefficient.

Now the density matrix has been evolved, we proceed to extract physical information from the output $\mathbf{v}_k(t) = \mathcal{N}_k \mathcal{M}_k \mathbf{v}_\rho$. Firstly the diagonal elements of each $\rho_k(t) = \mathbf{M}_k \rho \mathbf{M}_k^\dagger$ are always non-negative because $\mathbf{M}_k \rho \mathbf{M}_k^\dagger$ is positive-semidefinite. This implies that the diagonal elements of $\rho_k(t)$ can be obtained by applying a projection measurement on corresponding entries in $\mathbf{v}_k(t)$. Using an optical setup such as in Ref. [1] the probability of measuring each entry in $\mathbf{v}_k(t)$ can be efficiently obtained by recording the photon distribution at the output of the optical modes. Adding the diagonal elements of $\rho_k(t)$ over k gives us the diagonal elements of the final $\rho(t) = \sum_k \mathbf{M}_k \rho \mathbf{M}_k^\dagger$ which are the populations of the final system state in the basis currently in use.

Although the off-diagonal elements of $\rho_k(t)$ cannot be directly obtained without quantum tomography, they are nonetheless carried by $\mathbf{v}_k(t)$ and can become physically important. For example, if we want to obtain the populations of the final system in another basis, we can carry out a basis transformation $\rho_k(t) \to \mathbf{T}\rho_k(t)\mathbf{T}^\dagger$, or correspondingly $\mathbf{v}_k(t) \to (\mathbf{I}\otimes\overline{\mathbf{T}})(\mathbf{T}\otimes\mathbf{I})\mathbf{v}_k(t)$, where the off-diagonal elements are required. Here $\mathbf{T}$ is unitary such that $\mathbf{T}\otimes\mathbf{I}$ and $\mathbf{I}\otimes\overline{\mathbf{T}}$ are unitary, and therefore no dilations are needed. For the additional $\mathbf{T}\otimes\mathbf{I}$ and $\mathbf{I}\otimes\overline{\mathbf{T}}$ gates, the quantum gate count is increased by $n^3 - n^2$ to a total of $4n^3$ for each k, while the classical complexity adds an overhead cost of $4n^3 - 2n^2$ for $\rho(t) \to \mathbf{T}\rho(t)\mathbf{T}^\dagger$ (independent from the k count) to the original $4n^3 - 2n^2$ for each k. We remark that when the total number of $\mathbf{M}_k$ operators is small, the quantum algorithm outperforms the classical one by taking advantage of the unitarity of $\mathbf{T}$. The off-diagonal elements of $\rho_k(t)$ are also important if we want to calculate the expectation value of an observable: $\langle\mathbf{O}\rangle = Tr(\mathbf{O}\rho(t)) = \sum_k Tr(\mathbf{O}\rho_k(t))$. Here we use the same procedure as in the main text to define an operator $\tilde{\mathbf{O}} = \frac{\mathbf{O} + \mathbf{I}\|\mathbf{O}\|_{HS}}{2\|\mathbf{O}\|_{HS}}$, which is both a contraction and positive-semidefinite. Now by Cholesky decomposition we have $\tilde{\mathbf{O}} = \mathbf{L}\mathbf{L}^\dagger$ and:

$$\langle\tilde{\mathbf{O}}\rangle = Tr(\tilde{\mathbf{O}}\rho(t)) = Tr(\mathbf{L}\mathbf{L}^\dagger\rho(t)) = Tr(\mathbf{L}^\dagger\rho(t)\mathbf{L}) = \sum_k Tr(\mathbf{L}^\dagger\rho_k(t)\mathbf{L}) \tag{8}$$

where each $\mathbf{L}^\dagger\rho_k(t)\mathbf{L}$ is positive-semidefinite and its diagonal elements can be obtained by projection measurements. $\mathbf{L}^\dagger$ is obviously a contraction because $\tilde{\mathbf{O}}$ is a contraction. To realize

$$\mathbf{L}^\dagger\rho_k(t)\mathbf{L} \xleftrightarrow{equivalent} (\mathbf{I}\otimes\overline{\mathbf{L}}^\dagger)(\mathbf{L}^\dagger\otimes\mathbf{I})\mathcal{N}_k\mathcal{M}_k\mathbf{v}_\rho \tag{9}$$

we need all four matrices to be in the 4-dilation form:

$$\mathbf{U}_\mathbf{A} = \begin{pmatrix} \mathbf{A} & 0 & 0 & 0 & \mathbf{D}_{\mathbf{A}^\dagger} \\ \mathbf{D}_\mathbf{A} & 0 & 0 & 0 & -\mathbf{A}^\dagger \\ 0 & \mathbf{I} & 0 & 0 & 0 \\ 0 & 0 & \mathbf{I} & 0 & 0 \\ 0 & 0 & 0 & \mathbf{I} & 0 \end{pmatrix} \tag{10}$$

where $\mathbf{A} = (\mathbf{L}^\dagger\otimes\mathbf{I})$, $(\mathbf{I}\otimes\overline{\mathbf{L}}^\dagger)$, $\mathcal{N}_k$, or $\mathcal{M}_k$:

$$(\mathbf{I}\otimes\overline{\mathbf{L}}^\dagger)(\mathbf{L}^\dagger\otimes\mathbf{I})\mathcal{N}_k\mathcal{M}_k\mathbf{v}_\rho \xrightarrow{\text{unitary dilation}} \mathbf{U}_{(\mathbf{I}\otimes\overline{\mathbf{L}}^\dagger)}\mathbf{U}_{(\mathbf{L}^\dagger\otimes\mathbf{I})}\mathbf{U}_{\mathcal{N}_k}\mathbf{U}_{\mathcal{M}_k}(\mathbf{v}_\rho^T, 0, ..., 0)^T \tag{11}$$

Now after $\mathbf{L}^\dagger\rho_k(t)\mathbf{L}$ has been obtained $Tr(\tilde{\mathbf{O}}\rho(t))$ can be calculated, then we have:

$$
\begin{aligned}
\langle \mathbf{O} \rangle &= Tr\left(\mathbf{O}\rho(t)\right) \\
&= Tr\left(\left(2\|\mathbf{O}\|_{HS}\tilde{\mathbf{O}} - \mathbf{I}\|\mathbf{O}\|_{HS}\right)\rho(t)\right) \\
&= 2\|\mathbf{O}\|_{HS} Tr\left(\tilde{\mathbf{O}}\rho(t)\right) - \|\mathbf{O}\|_{HS}
\end{aligned}
\tag{12}
$$

where we have successfully obtained $\langle \mathbf{O} \rangle$ for the original observable. The $\left(\mathbf{L}^{\dagger} \otimes \mathbf{I}\right)$ and $\left(\mathbf{I} \otimes \bar{\mathbf{L}}^{\dagger}\right)$ gates ($\mathbf{L}^{\dagger}$ is upper triangular requiring reduced number of two-level unitaries for the decomposition) plus the additional two levels of dilation for $\mathcal{M}_k$ and $\mathcal{N}_k$ increase the quantum gate count to $5n^3 + 11n^2$ for each k. Calculating $\|\mathbf{O}\|_{HS}$ requires $2n^2 - 1$ classical arithmetic steps. The Cholesky decomposition has various implementations but generally it requires $\frac{n^3}{3}$ classical arithmetic steps[2]. Thus $\frac{n^3}{3} + 2n^2 - 1$ classical steps should be added as an overhead cost (independent from the k count) when evaluating the total cost of the quantum algorithm. In the meanwhile the classical complexity of evaluating $Tr\left(\mathbf{O}\rho(t)\right)$ adds an overhead of $2n^3 - n^2$ to the original $4n^3 - 2n^2$ for each k.

Next we demonstrate the method proposed above on the same amplitude damping model used in the main text. To calculate the populations in the current basis $\{|0\rangle, |1\rangle\}$ we can construct $\mathbf{U}_{\mathcal{M}_k}$ and $\mathbf{U}_{\mathcal{N}_k}$ of the 2-dilation form in Eq. (8) using $\mathcal{M}_k = \mathbf{M}_k \otimes \mathbf{I}$, $\mathcal{N}_k = \mathbf{I} \otimes \bar{\mathbf{M}}_k$, and $\mathbf{D}_{\mathbf{A}} = \sqrt{\mathbf{I} - \mathbf{A}^{\dagger}\mathbf{A}}$ with $\mathbf{A} = \mathcal{M}_k$, $\mathcal{N}_k$:

$$
\begin{aligned}
\mathcal{M}_0 &= \begin{pmatrix} 1 & 0 & 0 & 0 \\ 0 & 1 & 0 & 0 \\ 0 & 0 & \sqrt{e^{-\gamma t}} & 0 \\ 0 & 0 & 0 & \sqrt{e^{-\gamma t}} \end{pmatrix} \qquad
\mathcal{N}_0 = \begin{pmatrix} 1 & 0 & 0 & 0 \\ 0 & \sqrt{e^{-\gamma t}} & 0 & 0 \\ 0 & 0 & 1 & 0 \\ 0 & 0 & 0 & \sqrt{e^{-\gamma t}} \end{pmatrix} \\
\mathbf{D}_{\mathcal{M}_0} &= \begin{pmatrix} 0 & 0 & 0 & 0 \\ 0 & 0 & 0 & 0 \\ 0 & 0 & \sqrt{1-e^{-\gamma t}} & 0 \\ 0 & 0 & 0 & \sqrt{1-e^{-\gamma t}} \end{pmatrix} \qquad
\mathbf{D}_{\mathcal{N}_0} = \begin{pmatrix} 0 & 0 & 0 & 0 \\ 0 & \sqrt{1-e^{-\gamma t}} & 0 & 0 \\ 0 & 0 & 0 & 0 \\ 0 & 0 & 0 & \sqrt{1-e^{-\gamma t}} \end{pmatrix}
\end{aligned}
\tag{13}
$$

$$\mathcal{M}_1 = \begin{pmatrix} 0 & 0 & \sqrt{1-e^{-\gamma t}} & 0 \\ 0 & 0 & 0 & \sqrt{1-e^{-\gamma t}} \\ 0 & 0 & 0 & 0 \\ 0 & 0 & 0 & 0 \end{pmatrix} \qquad \mathcal{N}_1 = \begin{pmatrix} 0 & \sqrt{1-e^{-\gamma t}} & 0 & 0 \\ 0 & 0 & 0 & 0 \\ 0 & 0 & 0 & \sqrt{1-e^{-\gamma t}} \\ 0 & 0 & 0 & 0 \end{pmatrix}$$
$$\mathbf{D}_{\mathcal{M}_1} = \begin{pmatrix} 1 & 0 & 0 & 0 \\ 0 & 1 & 0 & 0 \\ 0 & 0 & \sqrt{e^{-\gamma t}} & 0 \\ 0 & 0 & 0 & \sqrt{e^{-\gamma t}} \end{pmatrix} \qquad \mathbf{D}_{\mathcal{N}_1} = \begin{pmatrix} 1 & 0 & 0 & 0 \\ 0 & \sqrt{e^{-\gamma t}} & 0 & 0 \\ 0 & 0 & 1 & 0 \\ 0 & 0 & 0 & \sqrt{e^{-\gamma t}} \end{pmatrix} \tag{14}$$

With an initial $\rho = \frac{1}{4}\begin{pmatrix} 1 & 1 \\ 1 & 3 \end{pmatrix}$, $\|\rho\|_{HS} = \frac{\sqrt{3}}{2}$, the input state is:

$$\mathbf{v}_0 = \frac{1}{\|\rho\|_{HS}}\left(\mathbf{v}_\rho^T, \overbrace{0,\ldots,0}^{m}\right)^T = \frac{1}{2\sqrt{3}}\left(1,1,1,3,\overbrace{0,\ldots,0}^{m}\right)^T \tag{15}$$

where $m = 8$ here is the number of zeros after $\mathbf{v}_\rho^T$. We use the same parameters as in the main text: set $\gamma = 1.52 \times 10^9 \mathrm{s}^{-1}$ (typical nanosecond lifetime), numerically calculate $\mathbf{U}_{\mathcal{N}_k}\mathbf{U}_{\mathcal{M}_k}\mathbf{v}_0$ from $t = 0$ to $t = 1000$ ps with a time step of 10 picosecond, and obtain the populations of the ground and excited states from the first and fourth entries of $\mathbf{U}_{\mathcal{N}_k}\mathbf{U}_{\mathcal{M}_k}\mathbf{v}_0$. The results are the same as the smooth lines in Figure 1 in the main text. To calculate the populations in another basis $\{|+\rangle, |-\rangle\}$ where $|\pm\rangle = \frac{1}{\sqrt{2}}(|0\rangle \pm |1\rangle)$, we need the transformation matrix $\mathbf{T} = \frac{1}{\sqrt{2}}\begin{pmatrix} 1 & 1 \\ 1 & -1 \end{pmatrix}$. Now numerically calculate $\begin{pmatrix} \mathbf{T}\otimes\mathbf{I} & & \\ & \mathbf{I} & \\ & & \mathbf{I} \end{pmatrix}\begin{pmatrix} \mathbf{I}\otimes\bar{\mathbf{T}} & & \\ & \mathbf{I} & \\ & & \mathbf{I} \end{pmatrix}\mathbf{U}_{\mathcal{N}_k}\mathbf{U}_{\mathcal{M}_k}\mathbf{v}_0$ and we can obtain the populations of the $|\pm\rangle$ states. The results are the same as the smooth lines in Figure 2 in the main text. Now we evaluate the expectation value of an observable $\langle\mathbf{O}\rangle$ for $\mathbf{O} = \begin{pmatrix} -2 & 0.5 \\ 0.5 & 1 \end{pmatrix}$ the same as used in the main text. We construct $\mathbf{U}_{(\mathbf{I}\otimes\bar{\mathbf{L}}^\dagger)}$, $\mathbf{U}_{(\mathbf{L}^\dagger\otimes\mathbf{I})}$, $\mathbf{U}_{\mathcal{M}_k}$ and $\mathbf{U}_{\mathcal{N}_k}$ of the 4-dilation form in Eq. (10) and apply them to the initial state $\mathbf{v}_0$ in the form of Eq. (15) with $m = 16$. Numerically calculating the output vector will give us $\langle\mathbf{O}\rangle$ by Eq. (12). The results are the same as the smooth line in Figure 3 in the main text.

4. **Quantum circuits used for the IBM Qiskit and Q 5 Tenerife device.**

To implement Method 2 on the IBM simulator and quantum device, we further decompose the unitary gates into 1-qubit and 2-qubit elementary gates and construct the quantum circuits. All the gates used below are standard. For each circuit, only the θ parameter of the $U_3=(\theta,\phi,\lambda)=\begin{pmatrix}\cos\frac{\theta}{2} & -e^{i\lambda}\sin\frac{\theta}{2}\\ e^{i\phi}\sin\frac{\theta}{2} & e^{i(\lambda+\phi)}\cos\frac{\theta}{2}\end{pmatrix}$ gate changes during the time evolution. The circuits below show the U_3 at the last time step at 991ps.

First for evolution in the original basis:

$\mathbf{U}_{\mathbf{M}_0}\mathbf{v}_1$:

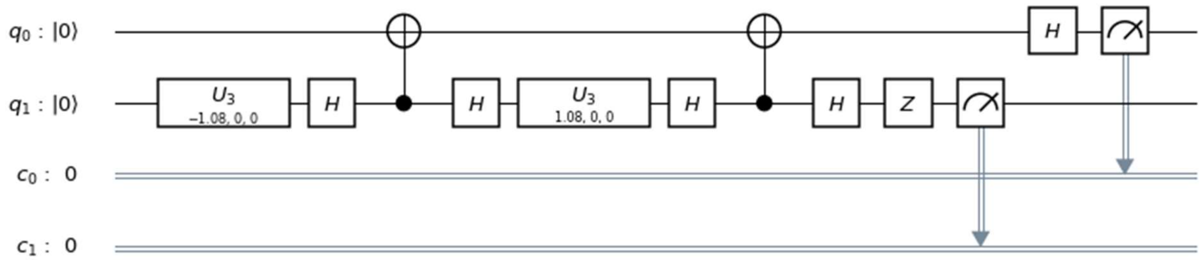

$\mathbf{U}_{\mathbf{M}_0}\mathbf{v}_2$:

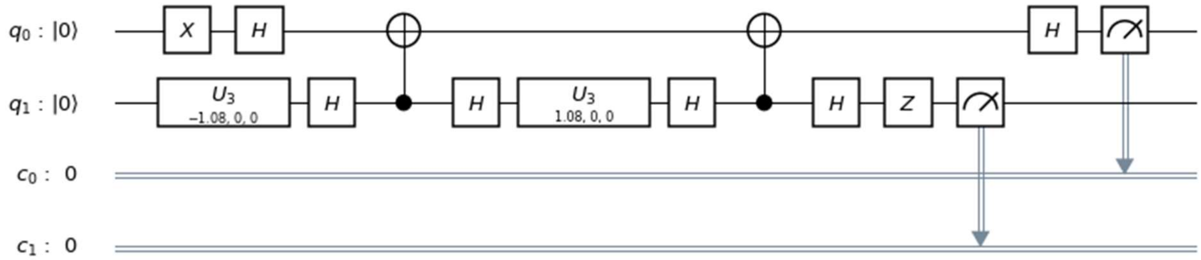

$\mathbf{U}_{\mathbf{M}_1}\mathbf{v}_1$:

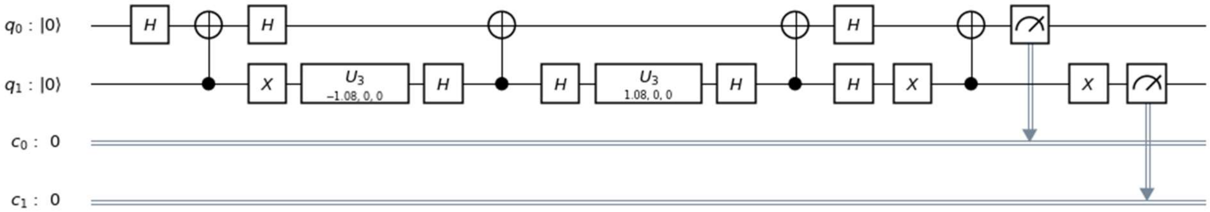

$\mathbf{U}_{\mathbf{M}_1}\mathbf{v}_2:$

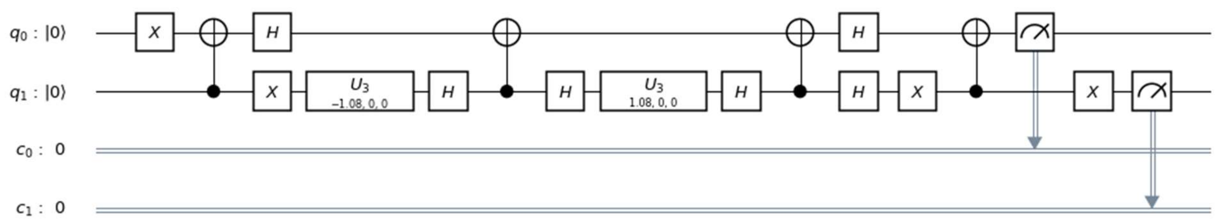

Next for evolution with a basis transformation:

$$\begin{pmatrix} \mathbf{T} & 0 \\ 0 & \mathbf{I} \end{pmatrix} \mathbf{U}_{\mathbf{M}_0}\mathbf{v}_1:$$

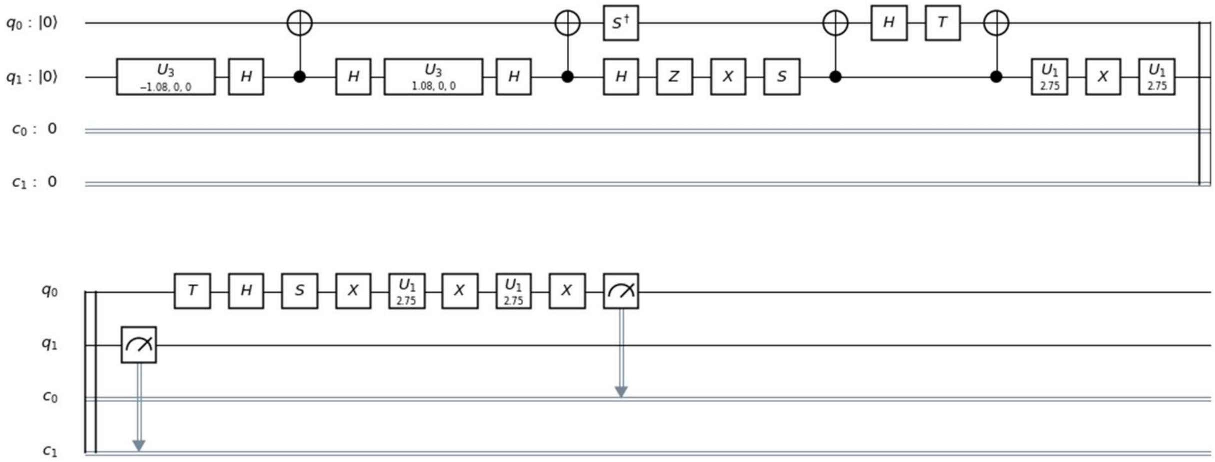

$$\begin{pmatrix} \mathbf{T} & 0 \\ 0 & \mathbf{I} \end{pmatrix} \mathbf{U}_{\mathbf{M}_0}\mathbf{v}_2:$$

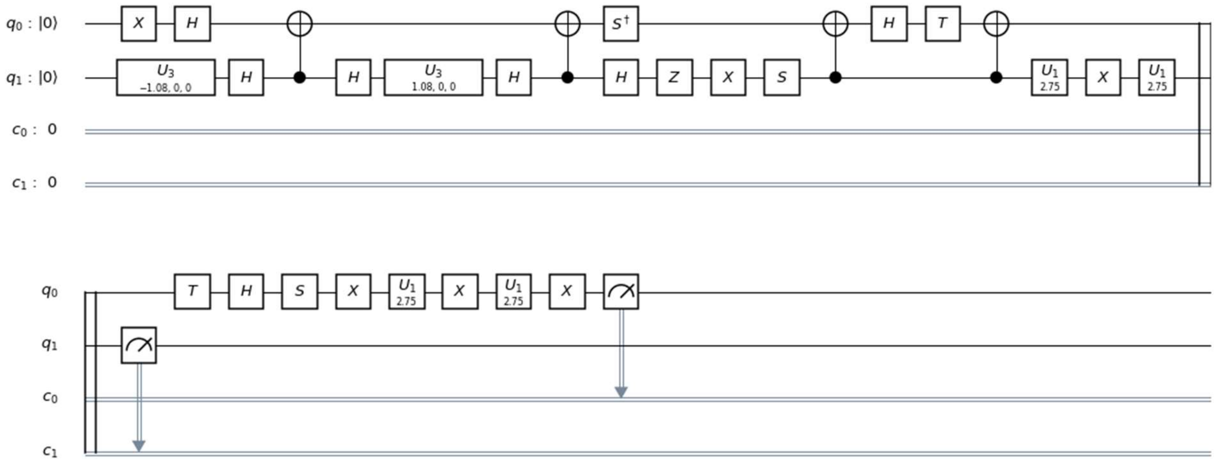

$\begin{pmatrix} \mathbf{T} & 0 \\ 0 & \mathbf{I} \end{pmatrix} \mathbf{U}_{\mathbf{M}_1} \mathbf{v}_1$:

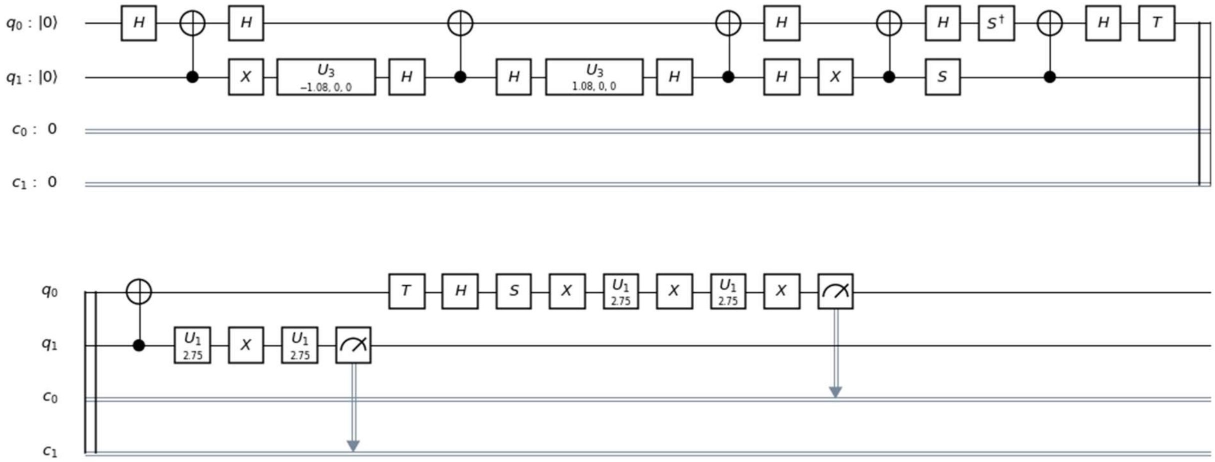

$\begin{pmatrix} \mathbf{T} & 0 \\ 0 & \mathbf{I} \end{pmatrix} \mathbf{U}_{\mathbf{M}_1} \mathbf{v}_2$:

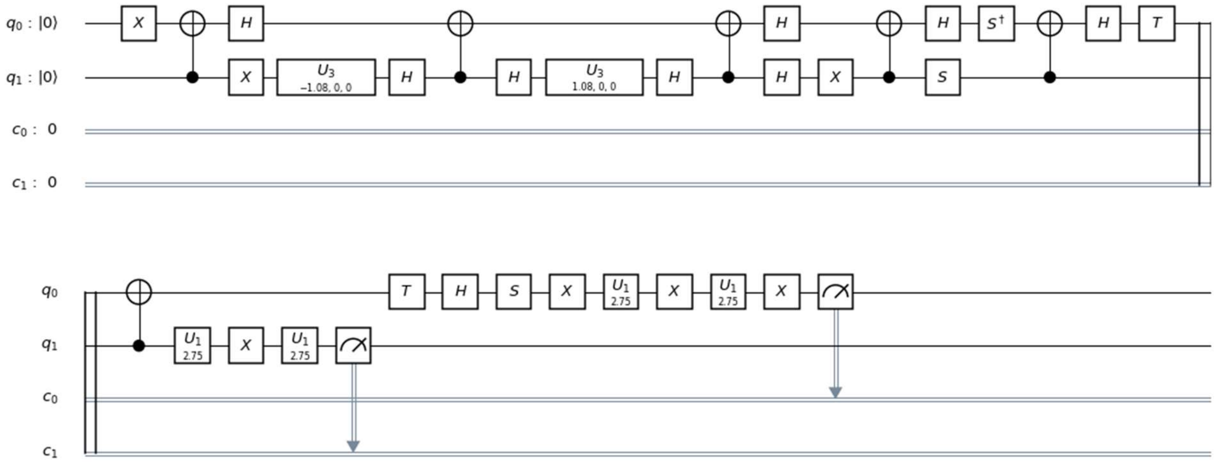

For the evolution with $\langle \tilde{\mathbf{O}} \rangle$ evaluation, due to the large number of gates involved we only show the circuit for $\mathbf{U}_{\mathbf{L}^\dagger} \mathbf{U}_{\mathbf{M}_0} \mathbf{v}_1$:

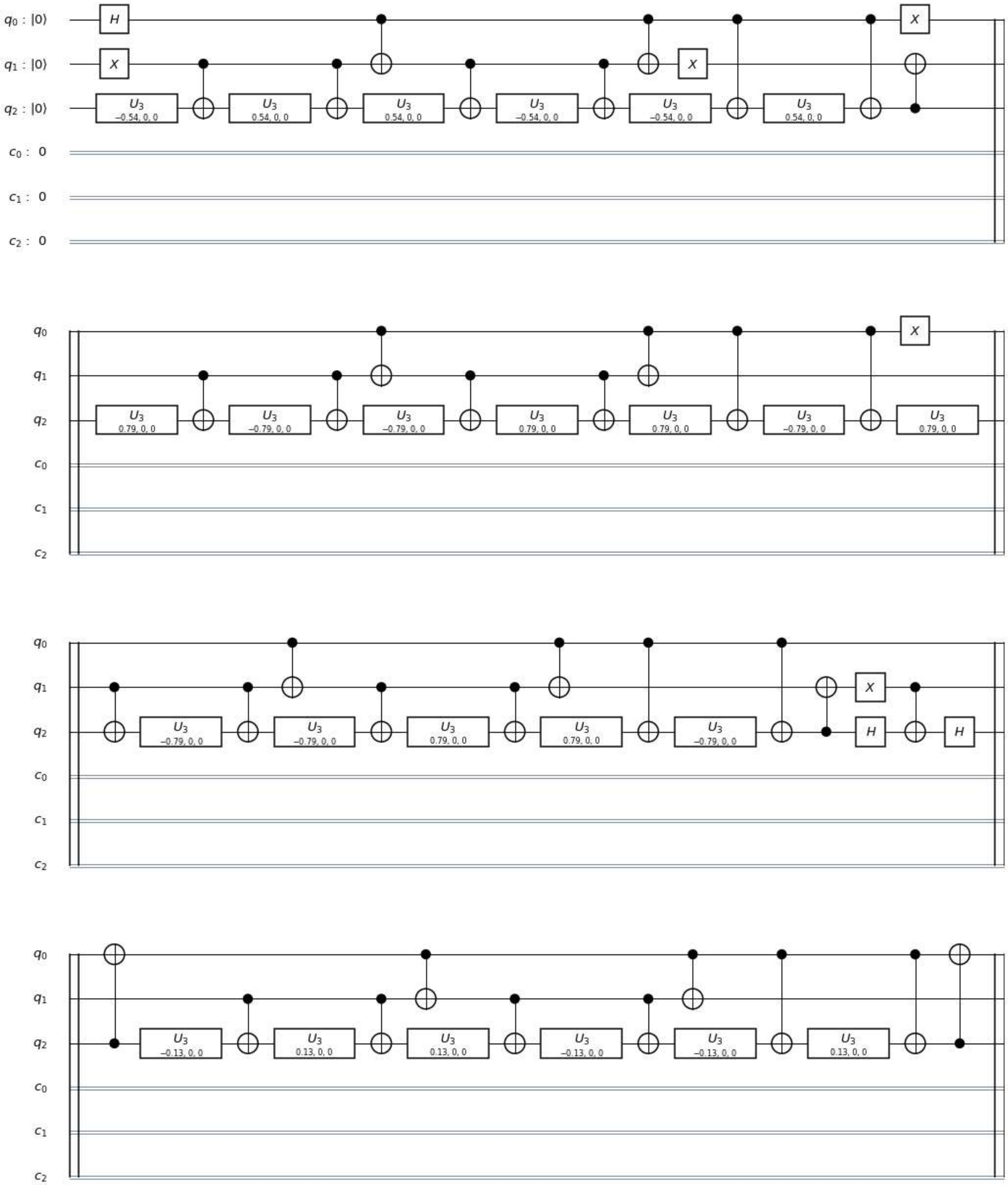
q0 : |0⟩
q1 : |0⟩
q2 : |0⟩
c0 : 0
c1 : 0
c2 : 0
H
X
U3
−0.54, 0, 0
0.54, 0, 0
0.54, 0, 0
−0.54, 0, 0
−0.54, 0, 0
0.54, 0, 0
X
X
q0
q1
q2
c0
c1
c2
U3
0.79, 0, 0
−0.79, 0, 0
−0.79, 0, 0
0.79, 0, 0
0.79, 0, 0
−0.79, 0, 0
0.79, 0, 0
X
q0
q1
q2
c0
c1
c2
U3
−0.79, 0, 0
−0.79, 0, 0
0.79, 0, 0
0.79, 0, 0
−0.79, 0, 0
X
H
H
q0
q1
q2
c0
c1
c2
U3
−0.13, 0, 0
0.13, 0, 0
0.13, 0, 0
−0.13, 0, 0
−0.13, 0, 0
0.13, 0, 0

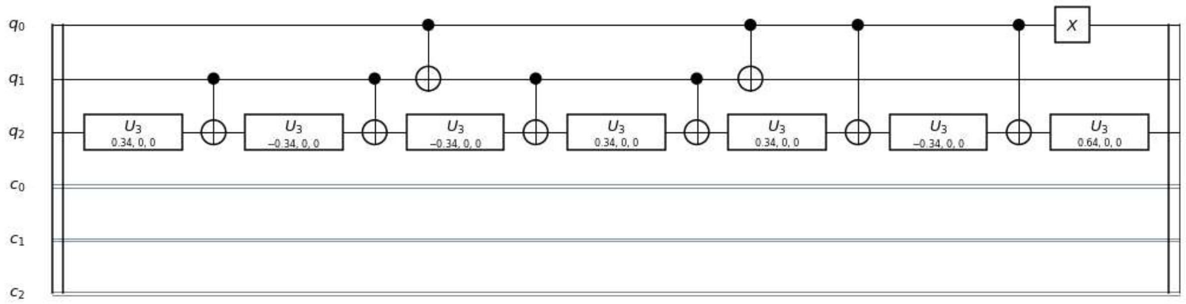
q0
q1
q2
c0
c1
c2
U3 0.34, 0, 0
U3 −0.34, 0, 0
U3 −0.34, 0, 0
U3 0.34, 0, 0
U3 0.34, 0, 0
U3 −0.34, 0, 0
U3 0.64, 0, 0
X
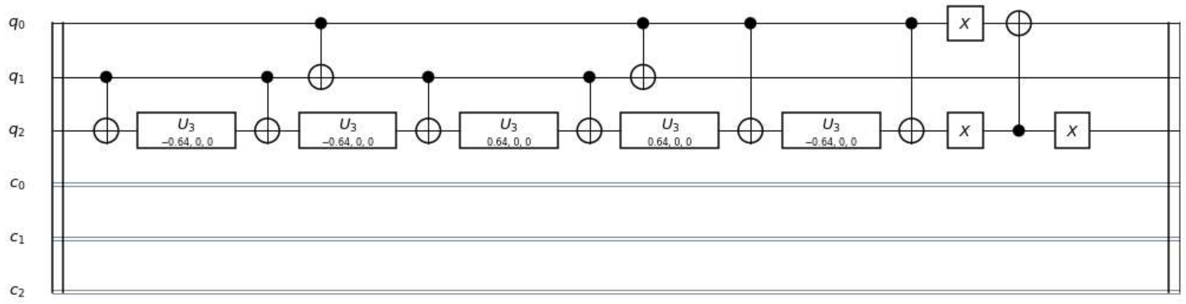
q0
q1
q2
c0
c1
c2
U3 −0.64, 0, 0
U3 −0.64, 0, 0
U3 0.64, 0, 0
U3 0.64, 0, 0
U3 −0.64, 0, 0
X
X
X
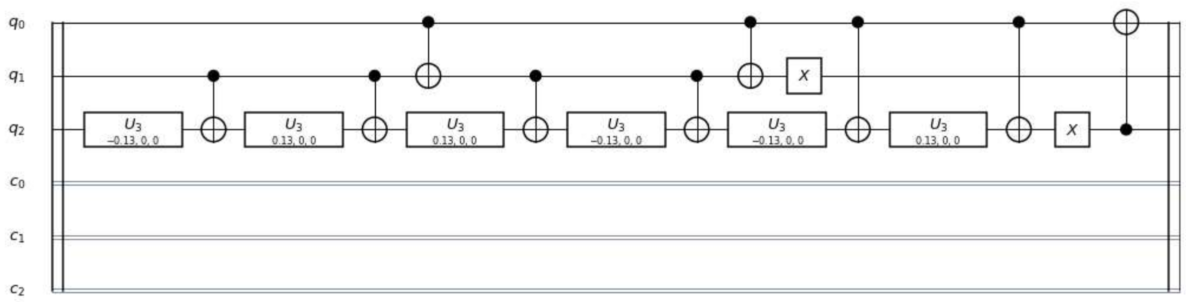
q0
q1
q2
c0
c1
c2
U3 −0.13, 0, 0
U3 0.13, 0, 0
U3 0.13, 0, 0
U3 −0.13, 0, 0
U3 −0.13, 0, 0
X
U3 0.13, 0, 0
X
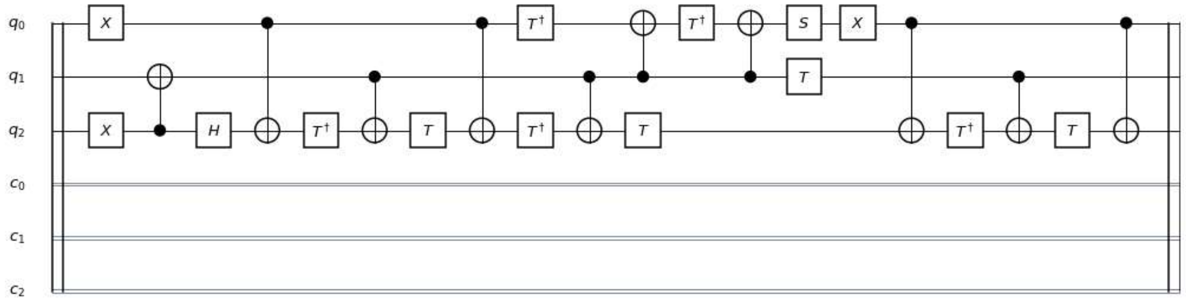
q0
q1
q2
c0
c1
c2
X
X
H
T†
T
T†
T†
T
T†
S
T
X
T†
T

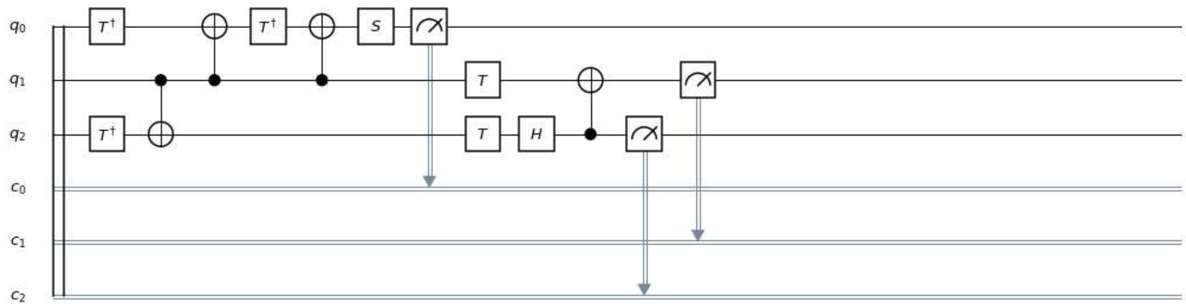

1. C. Sparrow, E. Martín-López, N. Maraviglia, A. Neville, C. Harrold, J. Carolan, Y. N. Joglekar, T. Hashimoto, N. Matsuda, J. L. O'Brien, D. P. Tew and A. Laing, *Nature*, 2018, **557**, 660-667.
2. A. Krishnamoorthy and D. Menon, 2013.
